# Comparative study of ChatGPT and human evaluators on the assessment of medical literature according to recognised reporting standards

**DOI:** 10.1136/bmjhci-2023-100830

**Published:** 2023-10-12

**Authors:** Richard HR Roberts, Stephen R Ali, Hayley A Hutchings, Thomas D Dobbs, Iain S Whitaker

**Affiliations:** 1 Reconstructive Surgery and Regenerative Medicine Research Centre, Swansea University, Swansea, UK; 2 Swansea University Medical School, Swansea University, Swansea, UK; 3 Welsh Centre for Burns and Plastic Surgery, Morriston Hospital, Swansea, UK

**Keywords:** Artificial intelligence, Medical Informatics

## Abstract

**Introduction:**

Amid clinicians’ challenges in staying updated with medical research, artificial intelligence (AI) tools like the large language model (LLM) ChatGPT could automate appraisal of research quality, saving time and reducing bias. This study compares the proficiency of ChatGPT3 against human evaluation in scoring abstracts to determine its potential as a tool for evidence synthesis.

**Methods:**

We compared ChatGPT’s scoring of implant dentistry abstracts with human evaluators using the Consolidated Standards of Reporting Trials for Abstracts reporting standards checklist, yielding an overall compliance score (OCS). Bland-Altman analysis assessed agreement between human and AI-generated OCS percentages. Additional error analysis included mean difference of OCS subscores, Welch’s t-test and Pearson’s correlation coefficient.

**Results:**

Bland-Altman analysis showed a mean difference of 4.92% (95% CI 0.62%, 0.37%) in OCS between human evaluation and ChatGPT. Error analysis displayed small mean differences in most domains, with the highest in ‘conclusion’ (0.764 (95% CI 0.186, 0.280)) and the lowest in ‘blinding’ (0.034 (95% CI 0.818, 0.895)). The strongest correlations between were in ‘harms’ (r=0.32, p<0.001) and ‘trial registration’ (r=0.34, p=0.002), whereas the weakest were in ‘intervention’ (r=0.02, p<0.001) and ‘objective’ (r=0.06, p<0.001).

**Conclusion:**

LLMs like ChatGPT can help automate appraisal of medical literature, aiding in the identification of accurately reported research. Possible applications of ChatGPT include integration within medical databases for abstract evaluation. Current limitations include the token limit, restricting its usage to abstracts. As AI technology advances, future versions like GPT4 could offer more reliable, comprehensive evaluations, enhancing the identification of high-quality research and potentially improving patient outcomes.

## Introduction

In the dynamic landscape of medical research, clinicians face the daunting challenge of staying abreast of the latest advancements amid their demanding clinical responsibilities. The rate and varying quality of emerging research further compounds this challenge. A number of appraisal tools exist to help readers assess the quality of the reported research, although these can also be time-consuming to employ and are at risk of user bias. The use of large language models (LLMs) like ChatGPT has the potential to automate this evaluation, thereby aiding clinicians in making informed decisions.^1^ However, the accuracy of LLMs compared with human expertise as a gold standard remains uncertain. In November 2023, OpenAI unveiled ChatGPT, a generative pretrained transformer (GPT) language model grounded in transformer architecture, which empowers it to process vast amounts of text data and generate coherent text outputs by discerning the relationships between input and output sequences. ChatGPT has been trained on extensive human language datasets, and several studies attest to its ability to produce high-quality, coherent text outputs.^2 3^ Clinical research applications of ChatGPT have yielded promising results, suggesting that artificial intelligence could potentially critically appraise abstracts and liberate valuable clinician time.^4^ The objective of this study is to compare the proficiency of ChatGPT3, the third iteration of OpenAI’s GPT model, in scoring abstracts against human evaluation as the benchmark. By determining the accuracy and efficiency of these LLMs in assessing research quality, we aim to explore their potential as valuable tools for clinicians in appraisal and evidence synthesis.

## Methods

**Figure 1:**
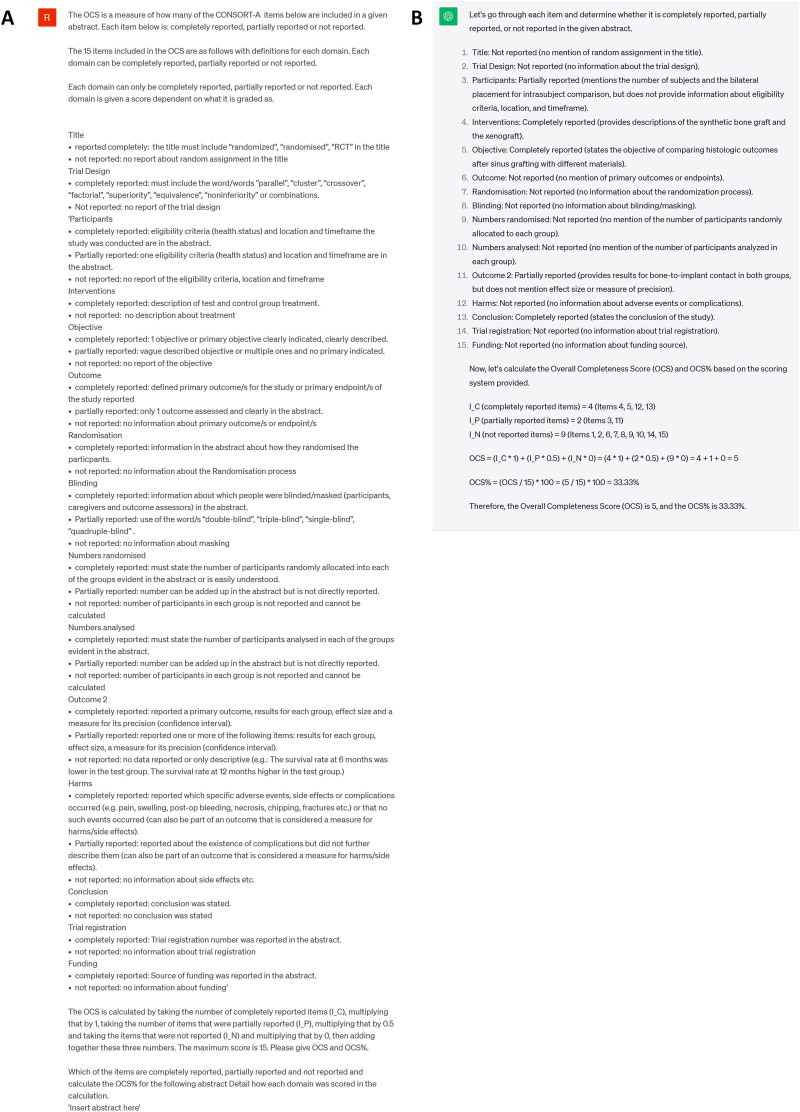
(A) Example prompt used to generate the OCS as per CONSORT-A criteria. (B) An example of the calculated OCS and OCS% as generated by ChatGPT. CONSORT-A, Consolidated Standards of Reporting Trials for Abstracts; OCS, overall compliance score.

In this study, we used a previously published paper as the basis of our comparison with ChatGPT.^5^ In their study, abstracts from a systematic review on implant dentistry were scored using the Consolidated Standards of Reporting Trials for Abstracts (CONSORT-A)^6^ statement by the human authors of the study. The processes of selection and data extraction were performed independently and in duplicate by two clinician reviewers across a sample of 30 abstracts. Discrepancies were systematically addressed through discussion until a consensus of at least 80% was achieved. Subsequent data extraction was conducted solely by one reviewer. The CONSORT-A checklist scores abstract reporting standards based on well-defined definitions for subsections such as trial design, blinding and randomisation. The human evaluators scored each item as fully reported, partially reported or not reported. ChatGPT was used to score the same set of abstracts, using a prompt to assess for each domain within the CONSORT-A checklist (figure 1). Building on the methodology established, each constituent subgroup was subsequently scored and categorised into one of the three classifications (figure 1A). An overall compliance score (OCS) was given out of 15, along with an OCS percentage (figure 1B). This was performed using the GPT3.5 model.

Bland-Altman analysis was used to evaluate the overall agreement between human and ChatGPT-generated OCS percentage. For error analysis, the mean difference of the absolute OCS subscores, Welch’s two-sample t-test and Pearson’s correlation coefficient were undertaken. The mean difference provides information on the magnitude and direction of the differences in OCS between ChatGPT and human evaluators, while the Pearson’s correlation coefficient provides information on the strength and direction of the linear relationship between the two sets of scores. This provided complementary information on the agreement between ChatGPT and human evaluator. The Pearson’s correlation coefficient was interpreted based on magnitude: r, 0–0.19 very weak, 0.2–0.39 weak, 0.40–0.59 moderate, 0.6–0.79 strong and 0.8–1 very strong correlation. Statistical analysis was done in R (V.4.1.1). P<0.001 was deemed statistically significant.

## Results

**Figure 2:**
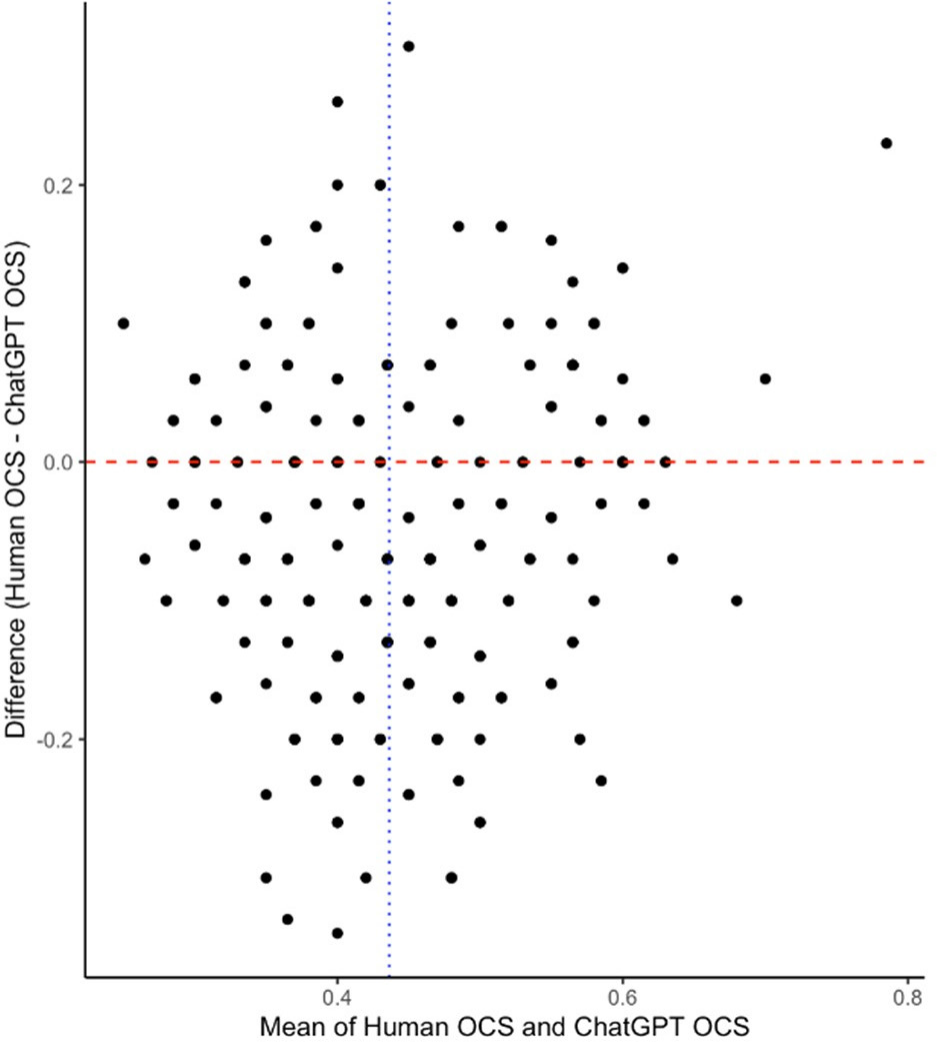
Bland-Altman analysis between ChatGPT human evaluation. OCS, overall compliance score.

Bland-Altman analysis revealed a mean difference of 4.92% (95% CI 0.62%, 0.37%) in OCS percentage (figure 2). Error analysis revealed small mean differences between human evaluation and ChatGPT in most domains (table 1).

**Table 1:** Error analysis of ChatGPT CONSORT-A OCS subscores

CONSORT-A domains	Mean difference in absolute OCS	P value*	Pearson’s correlation coefficient (r)
Trial design	0.065, 95% CI (0.579, 0.686)	0.054	0.49
Participants	0.228, 95% CI (0.485, 0.595)	0.001	0.26
Intervention	0.057, 95% CI (0.800, 0.881)	0.001	0.02
Objective	0.316, 95% CI (0.280, 0.384)	0.001	0.06
Outcome (methods)	0.553, 95% CI (0.077, 0.146)	0.001	0.14
Randomisation	0.633, 95% CI (0.277, 0.381)	0.001	0.11
Blinding	0.034, 95% CI (0.818, 0.895)	0.091	0.44
Number randomly assigned	0.105, 95% CI (0.530, 0.639)	0.006	0.31
Number analysed	0.028, 95% CI (0.475, 0.586)	0.434	0.04
Outcome (reporting)	0.170, 95% CI (0.453, 0.563)	0.001	0.15
Harms	0.133, 95% CI (0.602, 0.708)	0.001	0.32
Conclusion	0.764, 95% CI (0.186, 0.280)	0.001	0.06
Trial registration	0.045, 95% CI (0.918, 0.968)	0.002	0.34
Funding	0.411, 95% CI (0.533, 0.642)	0.001	0.21

*Welch’s two-sample t-test.

CONSORT-A, Consolidated Standards of Reporting Trials for Abstracts; OCS, overall compliance score.

The mean difference in absolute OCS was highest for the ‘conclusion’ domain (0.764, 95% CI: 0.186, 0.280), indicating that ChatGPT differed the most from human evaluators in this domain. In contrast, the domain with the lowest mean difference in absolute OCS was ‘blinding’ (0.034, 95% CI: 0.818, 0.895), indicating that ChatGPT was most accurate in this domain. In terms of correlation, the study found varying levels of correlation between ChatGPT and human evaluators for different domains. For example, the domains with a strong positive correlation were ‘harms’ (r=0.32, p<0.001) and ‘trial registration’ (r=0.34, p=0.002), indicating a high level of consistency between ChatGPT and human evaluators in these domains. On the other hand, ‘intervention’ (r=0.02, p<0.001) and ‘objective’ (r=0.06, p<0.001) domains had very weak correlations, suggesting that ChatGPT’s performance was less consistent with human evaluators in these domains.

## Discussion

The emergence of LLMs like ChatGPT offers a promising solution to streamline the assessment of reporting standards in medical literature and assist clinicians to make informed decisions. Bland-Altman analysis supports the overall findings of the study that ChatGPT has the potential to automate appraisal of medical literature. By providing a score for the quality of reporting in abstracts, ChatGPT can help clinicians and researchers quickly identify studies with more comprehensive and transparent reporting. The recent release of ChatGPT4, an advancement on the ChatGPT3 architecture, has demonstrated enhanced performance across diverse domains.^7 8^ Full access is currently limited by a paywall; however, its web integration technology creates immediate possibilities for further application. This could include searching for papers with minimum CONSORT compliance scores or the use of ChatGPT as a widget within popular medical databases, where it could automatically evaluate the quality of abstracts and provide a score to users promoting comprehensive and transparent reporting. One important barrier to using LLMs more widely in medical literature evaluation is the token limit. ChatGPT’s current token limit may not allow it to process the entire research articles, limiting its use to abstracts. Nevertheless, the potential to feed ChatGPT full papers in the future and have it evaluate studies using other appraisal tools is an exciting possibility. Large, unexpected differences were seen in the conclusion and outcome (methods) subdomains. In the context of LLMs such as ChatGPT, the paucity of data in relation to training makes pinpointing a singular cause challenging. However, the quality of the prompt has been underscored as a major determinant in response accuracy,^9^ and in the context of academic writing and interpretation, ChatGPT has been shown to not follow directions correctly.^10^ These may have played a pivotal role in the observed significant difference. Furthermore, some specifics of human evaluation were not elaborated upon and human assessment inaccuracies may have influenced scoring. Future research could cater to the assessment of variations between human evaluators and pave the way for a more in-depth analysis in conjunction with ChatGPT.

## Conclusion

As the technology continues to evolve and improve, the next iteration of GPT, GPT4, may further enhance the accuracy and efficiency of the tool, allowing for even more reliable and comprehensive evaluations of research. While there are still limitations to this technology, the promise it holds for assisting in the evaluation and identification of high-quality research is a significant step towards improving patient care and outcomes.
